# Supplementary material for: CTLA-4 expression by B-1a B cells is essential for immune tolerance
Source: Nat Commun. 2021 Jan 22;12:525. doi: 10.1038/s41467-020-20874-x (PMC7822855; doi:10.1038/s41467-020-20874-x)
Supplement: Supplementary file 1 — Supplementary Information [file 41467_2020_20874_MOESM1_ESM.pdf]

Supplementary Information

CTLA-4 expression by B-1a B cells is essential for immune tolerance

Yang et al

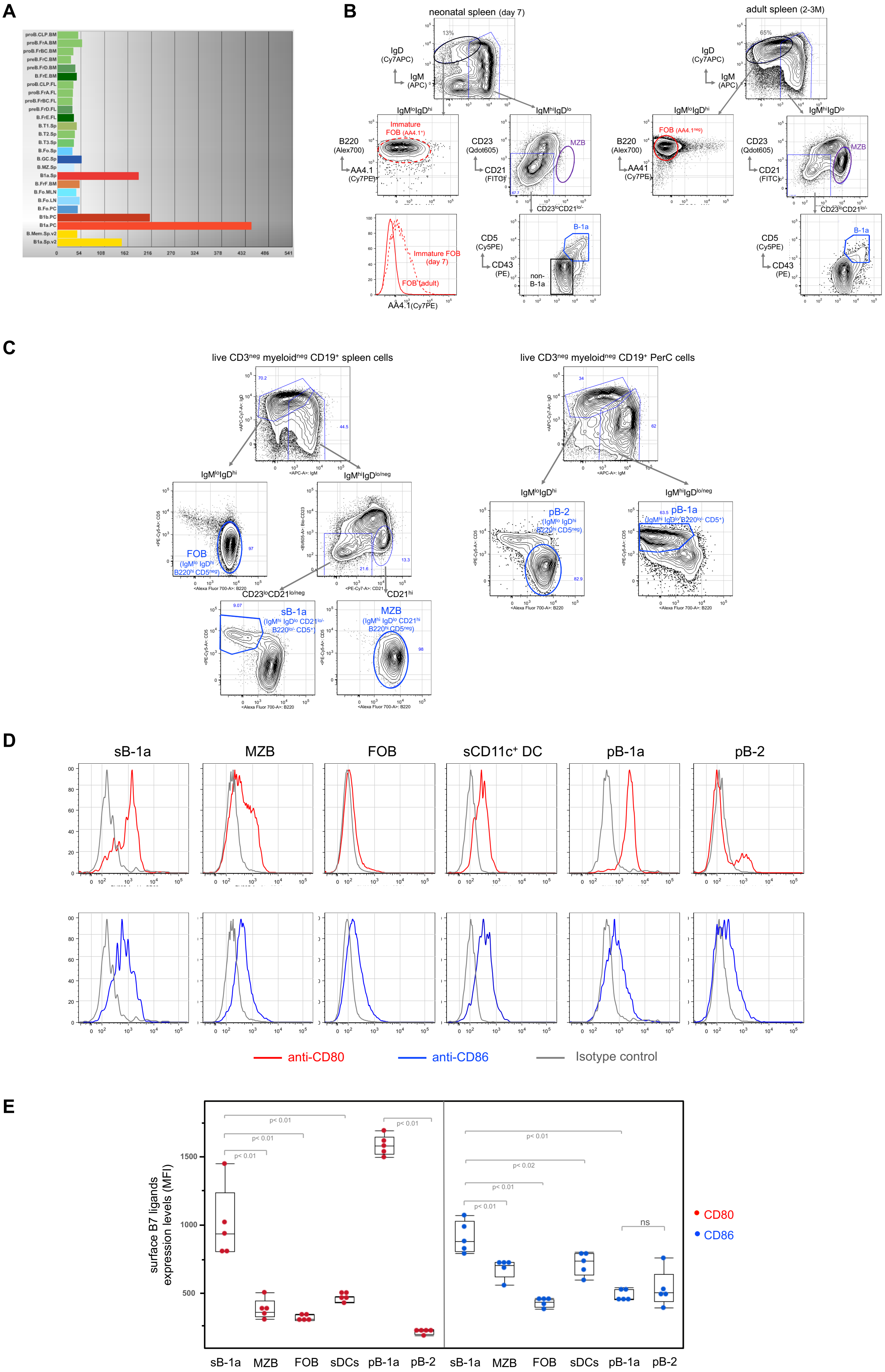

A
proB.CLP.BM
proB.FrA.BM
proB.FrBC.BM
preB.FrC.BM
preB.FrD.BM
B.FrE.BM
proB.CLP.FL
proB.FrA.FL
proB.FrBC.FL
preB.FrD.FL
B.FrE.FL
B.T1.Sp
B.T2.Sp
B.T3.Sp
B.Fo.Sp
B.GC.Sp
B.MZ.Sp
B1a.Sp
B.FrF.BM
B.Fo.MLN
B.Fo.LN
B.Fo.PC
B1b.PC
B1a.PC
B.Mem.Sp.v2
B1a.Sp.v2
0 54 108 162 216 270 324 378 432 486 541
B
neonatal spleen (day 7)
adult spleen (2-3M)
13%
65%
IgD (Cy7APC)
IgM (APC)
IgMloIgDhi
IgMhiIgDlo
Immature FOB (AA4.1+)
FOB (AA4.1neg)
B220 (Alex700)
AA4.1 (Cy7PE)
CD23 (Qdot605)
CD21 (FITC)
MZB
CD23loCD21lo/-
CD5 (Cy5PE)
CD43 (PE)
B-1a
non-B-1a
Immature FOB (day 7)
FOB (adult)
AA4.1(Cy7PE)
C
live CD3neg myeloidneg CD19+ spleen cells
live CD3neg myeloidneg CD19+ PerC cells
70.2
44.5
34
62
IgMloIgDhi
IgMhiIgDlo/neg
FOB (IgMlo IgDhi B220hi CD5neg)
97
21.6
13.3
CD23loCD21lo/neg
CD21hi
sB-1a (IgMhi IgDlo CD21lo/- B220lo/- CD5+)
9.07
MZB (IgMhi IgDlo CD21hi B220hi CD5neg)
98
pB-2 (IgMlo IgDhi B220hi CD5neg)
82.9
pB-1a (IgMhi IgDlo B220lo/- CD5+)
63.5
<Alexa Fluor 700-A>: B220
<PE-Cy5-A>: CD5
D
sB-1a
MZB
FOB
sCD11c+ DC
pB-1a
pB-2
anti-CD80
anti-CD86
Isotype control
E
surface B7 ligands expression levels (MFI)
500
1000
1500
p< 0.01
p< 0.02
ns
CD80
CD86
sB-1a
MZB
FOB
sDCs
pB-1a
pB-2

Supplementary Figure 1: B-1a cells constitutively express CTLA-4 and its B7 (CD80 and CD86) ligands

(A) Microarray data shows *Ctla4* expression by B cell subsets in C57BL/6J mice. Data was cited from Immunological Genome Project (ImmGen), a public resource and its data is publicly accessible through http://www.immgen.org/databrowser/index.html.

(B) FACS plots show the B cell subsets in spleen of neonate (day 7) and adult C57BL/6J mouse (2-3 months old). Live splenic CD19^{+} B cells were sequentially gated to show FOB (IgM^{lo} IgD^{hi} B220^{hi} AA4.1^{neg}), MZB (IgM^{hi} IgD$^{lo/neg}$ CD21^{hi}) and B-1a (IgM^{hi} IgD$^{lo/neg}$ CD21$^{lo/neg}$ CD23$^{lo/neg}$ CD43^{+} CD5^{+}) cells. A small proportion of IgM^{lo} IgD^{hi} B220^{hi} B cells is detected in day-7 neonatal spleen. They express AA4.1 (CD93) and are immature FOB cells.

(C) FACS plots show the gating strategy to define mature B cell subsets in intracellular CTLA-4 FACS analysis. FOB and peritoneal B-2 cells (pB-2), IgM^{lo} IgD^{hi} B220^{hi} CD5^{neg}; sB-1a, IgM^{hi} IgD$^{lo/neg}$ CD21$^{lo/neg}$ CD23$^{lo/neg}$ B220^{lo} CD5^{+}; pB-1a, IgM^{hi} IgD$^{lo/neg}$ B220^{lo} CD5^{+}; MZB, IgM^{hi} IgD$^{lo/neg}$ CD21^{hi} B220^{hi} CD5^{neg}.

(D) FACS histograms show the CD80 (red line) and CD86 (blue line) surface expression by indicated immune cells. Grey line shows data of cells stained with isotype control antibody conjugated with the same fluorochrome as anti-CD80 and anti-CD86 antibodies. sB-1a, IgM^{hi} IgD$^{lo/neg}$ CD21$^{lo/neg}$ CD5^{+} CD43^{+}; MZB, IgM^{hi} IgD$^{lo/neg}$ CD21^{hi} CD5^{neg} CD43^{neg}; FOB, IgM^{lo} IgD^{hi} CD5^{neg} CD43^{neg}; splenic CD11c^{+} (sCD11c^{+}) DCs, CD19^{neg} CD3^{neg} CD11c^{+}; pB-1a, IgM^{hi} IgD$^{lo/neg}$ CD5^{+} CD43^{+}; pB-2, IgM^{lo}IgD^{hi} CD5^{neg} CD43^{neg}.

(E) Data summarizing CD80 (red dot) and CD86 (blue dot) surface levels expressed by indicated immune subsets is shown. Each dot represents data for an individual mouse, n=5 mice per group. The expression levels are defined as CD80 or CD86 MFI value adjusted to the isotype control MFI value of all samples. ns, not significant ($p<0.2$), nonparametric Wilcoxon one-way test. Box plots: box draws 75% (upper), 50% (center line) and 25% (down) quartile, the maxima and minima outliers are shown as top and bottom line, respectively.

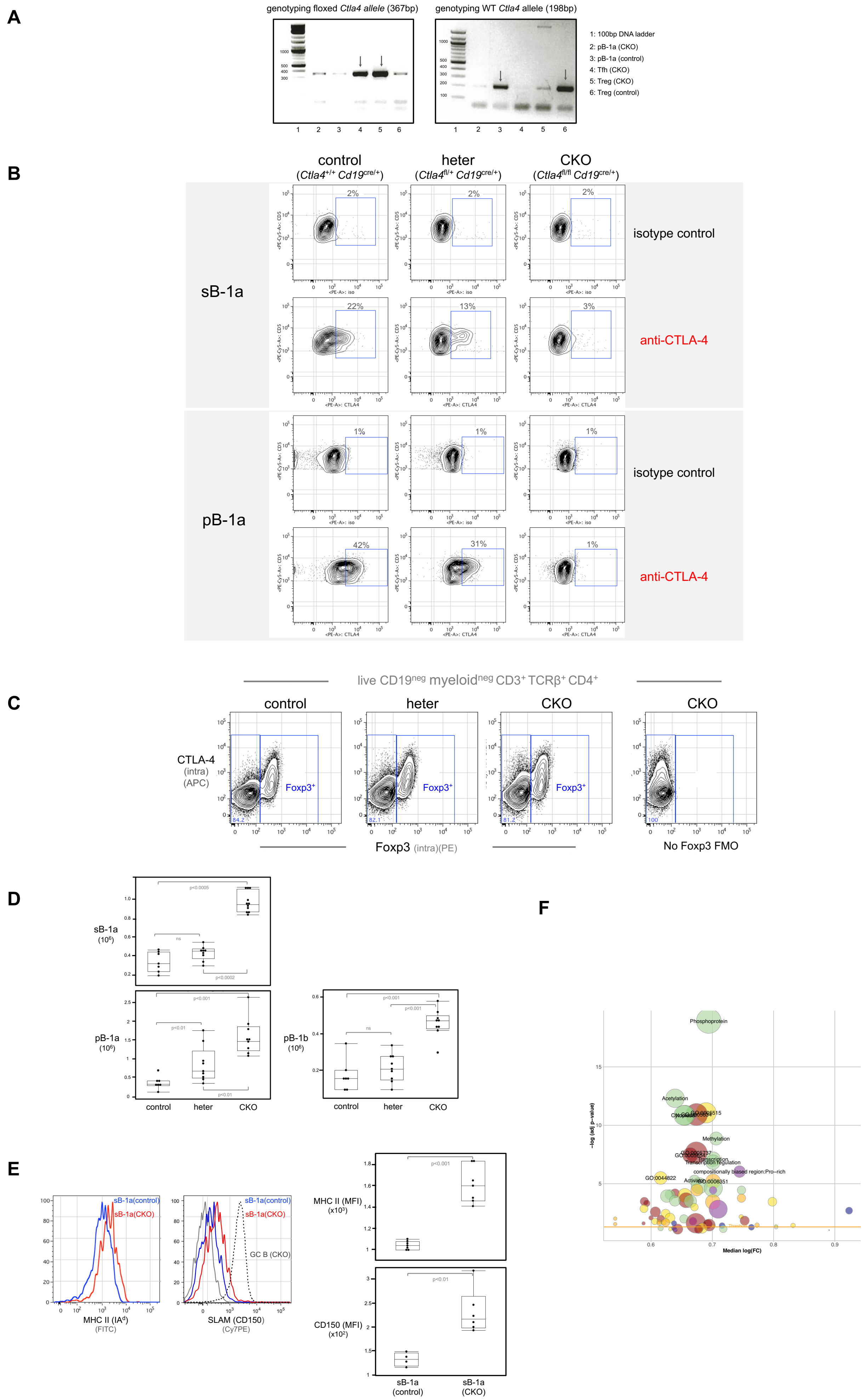
A
genotyping floxed Ctla4 allele (367bp)
genotyping WT Ctla4 allele (198bp)
1: 100bp DNA ladder
2: pB-1a (CKO)
3: pB-1a (control)
4: Tfh (CKO)
5: Treg (CKO)
6: Treg (control)
B
control (Ctla4+/+ Cd19cre/+)
heter (Ctla4fl/+ Cd19cre/+)
CKO (Ctla4fl/fl Cd19cre/+)
sB-1a
pB-1a
isotype control
anti-CTLA-4
2%
2%
2%
22%
13%
3%
1%
1%
1%
42%
31%
1%
C
live CD19neg myeloidneg CD3+ TCRβ+ CD4+
control
heter
CKO
CKO
CTLA-4 (intra) (APC)
Foxp3+
Foxp3 (intra)(PE)
No Foxp3 FMO
D
sB-1a (10⁶)
pB-1a (10⁶)
pB-1b (10⁶)
control
heter
CKO
p<0.0005
ns
p<0.0002
p<0.001
p<0.01
E
sB-1a(control)
sB-1a(CKO)
GC B (CKO)
MHC II (IAd) (FITC)
SLAM (CD150) (Cy7PE)
MHC II (MFI) (x10³)
CD150 (MFI) (x10²)
sB-1a (control)
sB-1a (CKO)
F
Phosphoprotein
Acetylation
Methylation
Transcription regulation
compositionally biased region:Pro-rich
GO:0044822
GO:0006351
−log (adj p-value)
Median log(FC)

Supplementary Figure 2: B-1a cells in CTLA-4 B cell CKO mice lose CTLA-4 expression and show increased activation.

(A) Peritoneal B-1a ($CD19^+$ IgM^{hi} $IgDl^{o/neg}$ $CD43^+$ $CD5^+$), Tfh ($CD3^+$ $CD4^+$ $PD\text{-}1^{hi}$ $CXCR5^+$), and Treg ($CD3^+$ $CD4^+$ $CD25^+$, >90% $Foxp3^+$) cells from control or CKO mice were sorted and their DNA were genotyped by PCR using two pairs of primers that detect floxed *Ctla4* and wild type (WT) *Ctla4* allele. Representative gel images show the PCR products for each subset. Arrows show the DNA fragments for the floxed (367bp) or WT *Ctla4* allele (198bp). Images of the uncropped gels are shown in the Source Data file.

(B) FACS plots show the intracellular CTLA-4 expression by splenic B-1a (sB-1a) and peritoneal B-1a (pB-1a) cells from indicated mice. FACS plots: *y*-axis shows surface CD5 expression and *x*-axis shows data for cells stained intracellularly with PE-conjugated anti-CTLA-4 antibody or isotype control antibodies.

(C) FACS plots show the intracellular CTLA-4 expression by splenic $Foxp3^+$ Treg cells from indicated mice. Live $CD4^+$ T cells were gated to reveal intracellular CTLA-4 and Foxp3 expression. The rightest FACS plot shows the fluorescent minus one (FMO) staining, in which the fluorescent anti-Foxp3 antibody was omitted from the staining cocktail to define Foxp3-expressing cells.

(D) Data summarizing numbers of sB-1a, pB-1a and pB-1b cells in control, heterozygous (heter) and CKO mice is shown. Each dot represents data for an individual mouse, n=7-11 mice per group, ns, not significant ($p<0.2$), nonparametric Wilcoxon one-way test.

(E) FACS histograms (left) show the MHC II (IA^d) and SLAM (CD150) surface expression on sB-1a ($CD19^+$ IgM^{hi} $IgD^{lo/neg}$ $CD23^{lo/neg}$ $CD43^+$ $CD5^+$) and GC B cells ($CD19^+$ $CD95^{hi}$ $CD38^{neg}$) from control and CKO mice. Data summarizing the MHC II and CD150 MFI value of sB-1a cells from control and CKO mice is shown (right), n=4-7 mice/group, nonparametric Wilcoxon one-way test. Box plots in D, E: box draws 75% (upper), 50% (center line) and 25% (down) quartile, the maxima and minima outliers are shown as top and bottom line, respectively.

(F) Graph shows the overview of the gene sets annotation enrichment analysis results for differentially expressed genes between splenic B-1a cells from control and CKO mice. *x*-axis represents the median fold change on logarithmic scale, and y-axis represents the negative logarithm of the adjusted p-value (the higher, the more significant). The area of the displayed circles is proportional to the number of genes assigned to the term. All the terms with the negative logarithm of the adjusted *p*-value larger than two were shown.

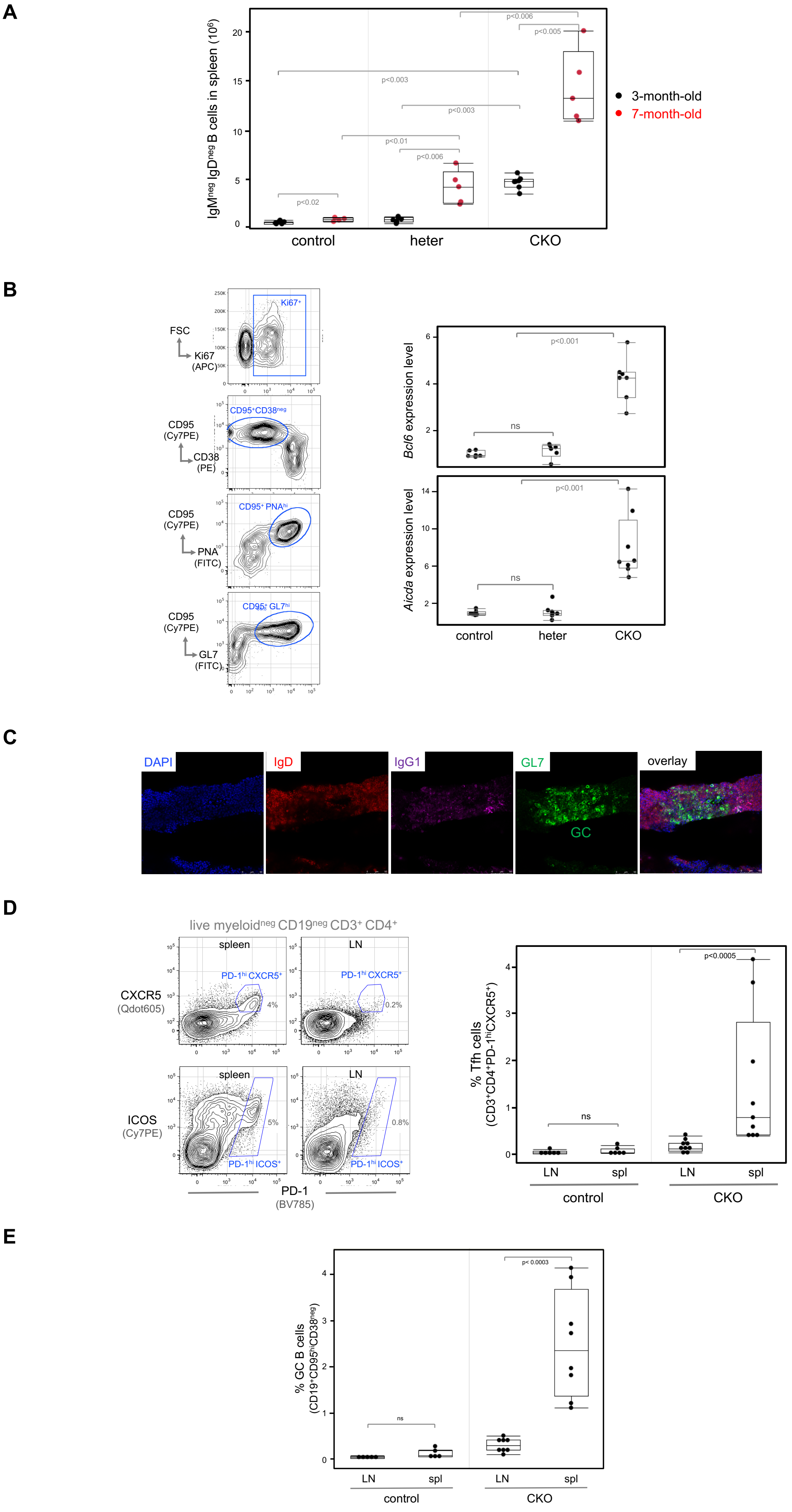
A
IgMneg IgDneg B cells in spleen (10^6)
p<0.006
p<0.005
p<0.003
p<0.003
p<0.01
p<0.006
p<0.02
3-month-old
7-month-old
control
heter
CKO
B
FSC
Ki67 (APC)
Ki67+
CD95 (Cy7PE)
CD38 (PE)
CD95+CD38neg
CD95 (Cy7PE)
PNA (FITC)
CD95+ PNAhi
CD95 (Cy7PE)
GL7 (FITC)
CD95+ GL7hi
Bcl6 expression level
p<0.001
ns
Aicda expression level
p<0.001
ns
control
heter
CKO
C
DAPI
IgD
IgG1
GL7
overlay
GC
D
live myeloidneg CD19neg CD3+ CD4+
spleen
LN
PD-1hi CXCR5+
4%
0.2%
CXCR5 (Qdot605)
ICOS (Cy7PE)
5%
0.8%
PD-1hi ICOS+
PD-1 (BV785)
% Tfh cells (CD3+CD4+PD-1hiCXCR5+)
p<0.0005
ns
LN
spl
LN
spl
control
CKO
E
% GC B cells (CD19+CD95hiCD38neg)
p< 0.0003
ns
LN
spl
LN
spl
control
CKO

Supplementary Figure 3: CTLA-4 B cell CKO mice spontaneously generate GCs and Tfh cells in the spleen.

(A) Data summarizing numbers of $CD19^+$ IgM^{neg} IgD^{neg} cells in spleens of 3 or 7-month-old control, heter and CKO mice is shown. Each dot represents data for an individual mouse, n=5-6 mice per group, nonparametric Wilcoxon one-way test.

(B) FACS plots on the left show IgM^{neg} IgD^{neg} B cells in spleen of CKO mice express GC B cell markers. Live $CD19^+$ IgM^{neg} IgD^{neg} splenic cells from CKO mice were gated to reveal CD95, Ki67, CD38, PNA and GL7 expression. $CD19^+$ IgM^{neg} IgD^{neg} cells in spleens of control, heter and CKO mice were sorted and their *Bcl6* and *Aicda* expression were measured by qRT-PCR. The right panel shows the data summarizing *Bcl6* and *Aicda* expression by IgM^{neg} IgD^{neg} B cells from each mouse line, n=6-7 independent samples per group, ns, not significant ($p<0.2$), nonparametric Wilcoxon one-way test.

(C) Spleen sections of CKO mice were analyzed by immunofluorescence confocal microscopy. Representative immunofluorescence images of the sections stained with indicated reagents and overlaid three-color composite are shown.

(D) Spleen and LN cells from CKO mice were analyzed by FACS. FACS plots (right): live $CD4^+$ T cells in spleen and LN of CKO mice were gated to reveal PD-1, CXCR5 and ICOS surface expression. Tfh cells, $PD\text{-}1^{hi}$ $CXCR5^+$ $ICOS^+$. Data summarizing the frequencies of Tfh cells in spleen and LN from control and CKO mice is shown (right), n=6-9 mice per group, ns, not significant ($p<0.4$), nonparametric Wilcoxon one-way test.

(E) Data summarizing the frequencies of GC B cells in spleen and LN from control and CKO mice is shown. Each dot represents data for an individual mouse, n=5-8 mice per group, ns, not significant ($p<0.1$), nonparametric Wilcoxon one-way test. Box plots in A, C, D-E: box draws 75% (upper), 50% (center line) and 25% (down) quartile, the maxima and minima outliers are shown as top and bottom line, respectively.

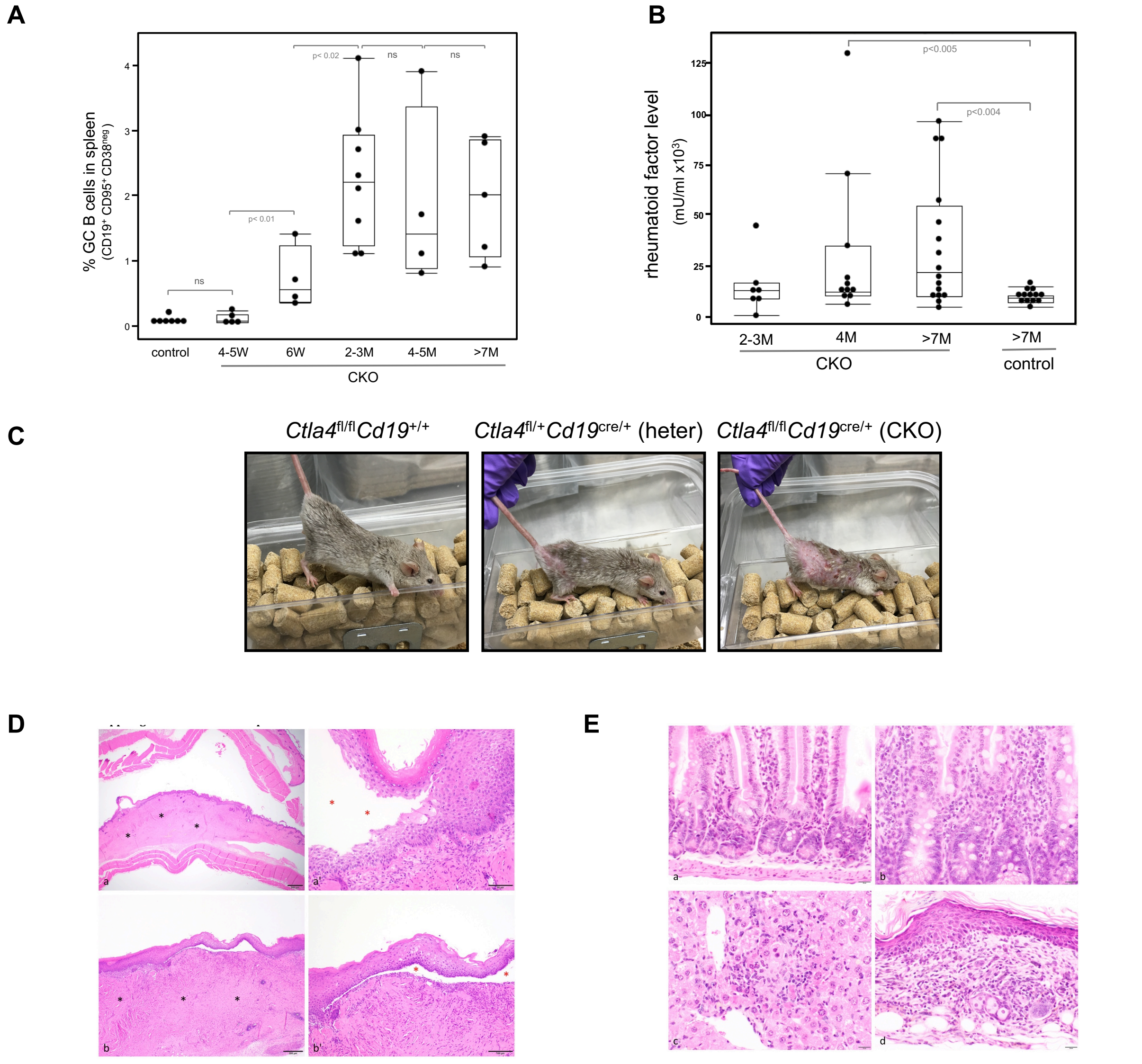

Supplementary Figure 4: CTLA-4 B cell CKO mice produce autoantibodies and develop autoimmune pathology in later life.

(A) Data summarizing the frequencies of GC B cells in spleens of control and CKO mice at indicated age is shown. Each dot represents data for an individual mouse, n=4-8 mice/group, ns, not significant ($p<0.4$), nonparametric Wilcoxon one-way test. W, week; M, month.

(B) Elisa assay shows the rheumatoid factor antibody levels in serum of control or CKO mice at the indicated age. Each dot represents data for an individual mouse, n=7-15 mice per group, nonparametric Wilcoxon one-way test.

(C) Pictures of three representative mice (7-month-old) are shown. They were derived from the same litter but expressed different genotype.

(D) Representative images of H&E stained skin sections from a CKO mouse show the cutaneous pathology. The dermis is occasionally expanded by dense and sometimes hyalinized eosinophilic matrix (black asterisks; a, b). In the overlying epidermal epithelium there is occasional intra-epidermal clefting (red asterisks, a'), or clefting at the dermal-epidermal junction (red asterisks, b'). a' is a higher magnification of a. b' is a higher magnification of b. Magnification: a= 1.25x; a'= 20x; b= 4x; b'= 10x Scale bar: a= 500µm; a'= 50µm; b= 200µm; b'= 100µm.

(E) Representative images of H&E stained sections from CKO mice show the inflammation in various organs. Inflammation in the intestine varied from mild (a) to moderate (b) and was composed of neutrophils and/or lymphocytes. Additional organs affected by inflammatory influx included the liver (c) and the skin of the ear (pinna; d) Magnification: a-d= 40x Scale bar: a-d= 20µm.

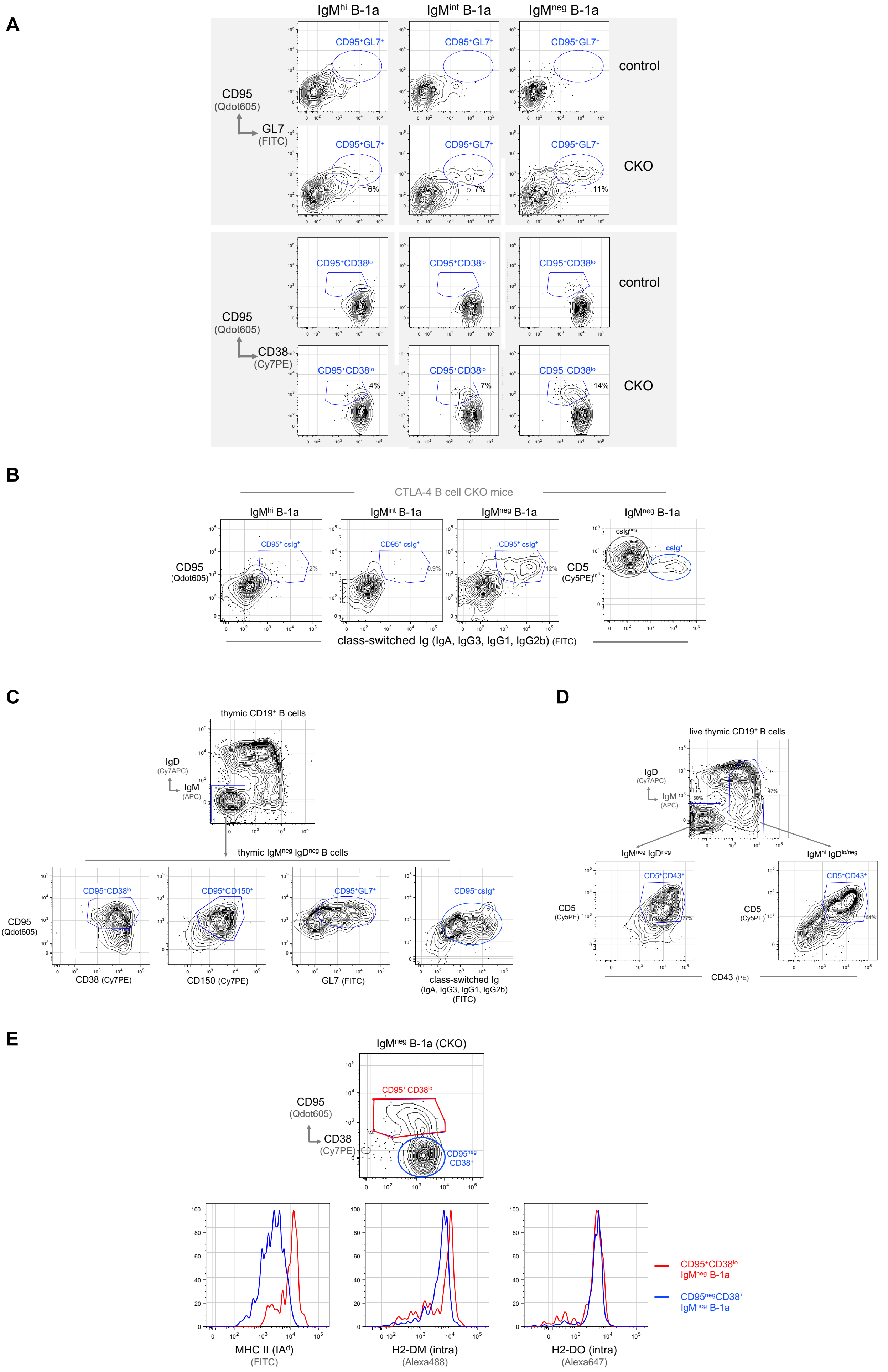
A
IgMhi B-1a
IgMint B-1a
IgMneg B-1a
CD95 (Qdot605)
GL7 (FITC)
CD95+GL7+
control
CKO
6%
7%
11%
CD38 (Cy7PE)
CD95+CD38lo
4%
7%
14%
B
CTLA-4 B cell CKO mice
CD95+ csIg+
2%
0.9%
12%
CD5 (Cy5PE)
csIgneg
csIg+
class-switched Ig (IgA, IgG3, IgG1, IgG2b) (FITC)
C
thymic CD19+ B cells
IgD (Cy7APC)
IgM (APC)
thymic IgMneg IgDneg B cells
CD95+CD38lo
CD95+CD150+
CD95+GL7+
CD95+csIg+
CD38 (Cy7PE)
CD150 (Cy7PE)
GL7 (FITC)
class-switched Ig (IgA, IgG3, IgG1, IgG2b) (FITC)
D
live thymic CD19+ B cells
30%
47%
IgMneg IgDneg
IgMhi IgDlo/neg
CD5+CD43+
77%
54%
CD43 (PE)
E
IgMneg B-1a (CKO)
CD95+ CD38lo
CD95neg CD38+
MHC II (IAd) (FITC)
H2-DM (intra) (Alexa488)
H2-DO (intra) (Alexa647)
CD95+CD38lo IgMneg B-1a
CD95negCD38+ IgMneg B-1a

F

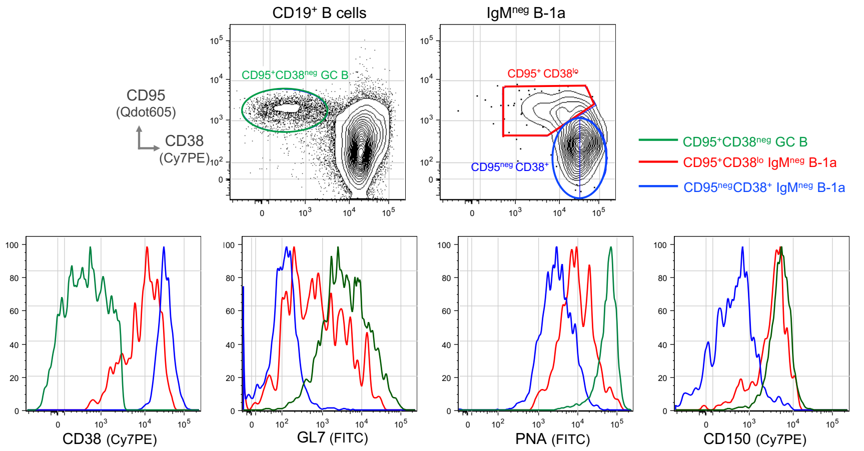

G

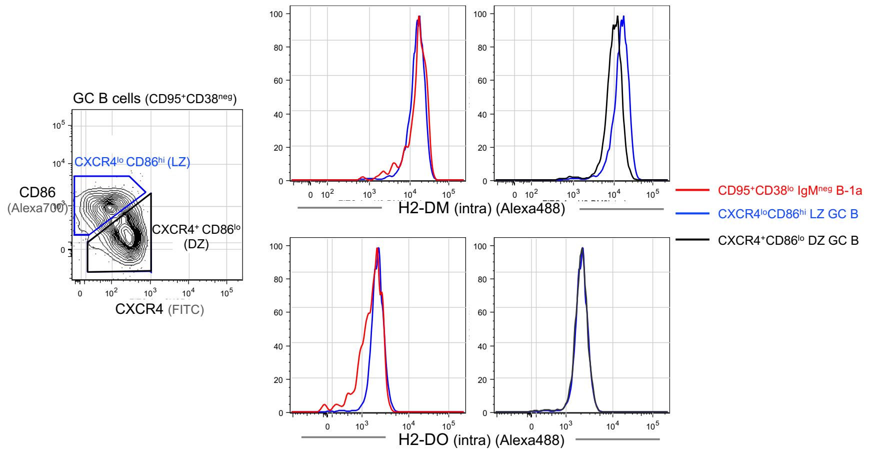

H

```
>M04151:96:000000000-BCB9N:1:1107:7434:10549 COPY:1 mIGHV11-2*02[0] mIGHD2-8*01 mIGHJ1*01[0] mIGHE*01 MRYGNYWYFDV
 T  P  G  K  T  L  E  W  I  G  D  I  N  S  D  G  S  A  I  N  Y  A  P  S  I  K  D  R  F  T  I  F  R  D  N  D  K  S  T  L
       .           .           .           .     †           .           .           .           .           ‡           .           .
acacctgggaagaccctggagtggattGGAGACATTAATTCTGATGGCAGTGCAATAAACTACGCACCATCCATAAAGGATCGATTCACTATCTTCAGAGACAATGACAAGAGCACCCTG
ACACCTGGGAAGACCCTGGAGTGGATTGGAGACATTAATTCTGATGGCAGTGCAATAAACTACGCACCATCCATAAAGGATCGATTCACTATCTTCAGAGACAATGACAAGAGCACCCTG

 Y  L  Q  M  S  N  V  R  S  E  D  T  A  T  Y  F  C  M  R  Y  G  N  Y  W  Y  F  D  V  W  G  A  G  T  S  V  T  A  S  S  A
       .           .           †           .           .           .           .           ‡           .           .           .           .
TACCTGCAGATGAGCAATGTGCGATCTGAGGACACAGCCACGTATTTCTGTATGAGATATGGTAACTACTGGTACTTCGATGTCTGGGGCGCAGGAACCTCAGTCACCGCCTCCTCAGCC
TACCTGCAGATGAGCAATGTGCGATCTGAGGACACAGCCACGTATTTCTGTATGAGATATGGTAACTACTGGTACTTCGATGTCTGGGGCGCAGGGACCACGGTCACCGTCTCCTCAGCC
```

```
>M04151:96:000000000-BCB9N:1:2107:16809:23871 COPY:1 mIGHV11-2*02[0] mIGHD1-1*02 mIGHJ1*01[0] mIGHG1*02 MRYGSYWYFDV
 T  P  G  K  T  V  E  W  I  G  D  I  N  S  D  G  S  A  I  N  Y  A  P  S  I  K  D  R  F  T  I  F  G  D  N  D  K  S  T  L
       .           .           .           .     †           .           .           .           .           ‡           .           .
acacctgggaagaccgtggagtggatTGGAGACATTAATTCTGATGGCAGTGCAATAAACTACGCACCATCCATAAAGGATCGATTCACTATCTTCGGAGACAATGACAAGAGCACACTG
ACACCTGGGAAGACCCTGGAGTGGATTGGAGACATTAATTCTGATGGCAGTGCAATAAACTACGCACCATCCATAAAGGATCGATTCACTATCTTCAGAGACAATGACAAGAGCACCCTG

 Y  L  Q  M  S  N  V  R  S  E  D  T  A  M  Y  F  C  M  R  Y  G  S  Y  W  Y  F  D  V  W  G  A  G  T  T  V  T  V  S  S  A
       .           .           †           .           .           .           .           ‡           .           .           .           .
TACCTGCAGATGAGCAATGTGCGATCTGAGGACACAGCCATGTATTTCTGTATGAGATATGGTAGCTACTGGTACTTCGATGTCTGGGGCGCAGGGACCACGGTCACCGTCTCCTCAGCC
TACCTGCAGATGAGCAATGTGCGATCTGAGGACACAGCCACGTATTTCTGTATGAGATATGGTAGCTACTGGTACTTCGATGTCTGGGGCGCAGGGACCACGGTCACCGTCTCCTCAGCC
```

```
>M04151:96:000000000-BCB9N:1:1107:8576:15179 COPY:1 mIGHV1-53*01[2] mIGHD4-1*02 mIGHJ3*01[0] mIGHA*08 ATGTWFAY
 R  P  G  Q  G  L  E  W  I  G  N  I  N  P  S  N  G  V  T  N  Y  N  E  K  F  K  I  K  V  T  L  T  V  D  K  S  S  S  T  A
       .           .           .           .     †           .           .           .           .           ‡           .           .
aggcctggacaaggccttgaGTGGATTGGAAATATTAATCCTAGCAATGGTGTTACTAACTACAATGAGAAGTTCAAGATCAAGGTCACTCTGACTGTAGACAAATCCTCCAGCACAGCC
AGGCCTGGACAAGGCCTTGAGTGGATTGGAAATATTAATCCTAGCAATGGTGGTACTAACTACAATGAGAAGTTCAAGAGCAAGGCCACACTGACTGTAGACAAATCCTCCAGCACAGCC

 Y  M  Q  L  S  S  L  T  S  E  D  S  A  V  Y  Y  C  A  T  G  T  W  F  A  Y  W  G  Q  G  T  L  V  T  V  S  A  A  K  T  T
       .           .           †           .           .           .           .           ‡           .           .           .           .
TACATGCAGCTCAGCAGCCTGACATCTGAGGACTCTGCGGTCTATTATTGTGCAACTGGGACCTGGTTTGCTTACTGGGGCCAAGGGACTCTGGTCACTGTCTCTGCAGCCAAAACGACA
TACATGCAGCTCAGCAGCCTGACATCTGAGGACTCTGCGGTCTATTATTGTGCAACTGGGACCTGGTTTGCTTACTGGGGCCAAGGGACTCTGGTCACTGTCTCTGCAG
```

Supplementary Figure 5: Activated B-1a cells in CTLA-4 B cell CKO mice turn off surface IgM and start expressing the pre-GC APC phenotype.

(A) FACS plots show cells expressing the differentiated $CD95^{+}$ $CD38^{lo}$ $GL7^{+}$ phenotype in IgM^{hi} B-1a, IgM^{int} B-1a and IgM^{neg} B-1a cells from control and CKO mice.

(B) FACS plots show cells expressing CD95 and surface class-switched Ig (csIg) on IgM^{hi} B-1a, IgM^{int} B-1a and IgM^{neg} B-1a cells from CKO mice. The FACS plot on the right shows that class-switched Ig ($csIg^{+}$) IgM^{neg} B-1a cells express reduced levels of CD5 than $csIg^{neg}$ IgM^{neg} B-1a cells.

(C) Cells of thymus from control mice were analyzed by FACS. Live B cells were gated to reveal IgM^{neg} IgD^{neg} B cells, which were further gated to show CD95, CD38, GL7, PNA and csIg expression.

(D) IgM^{neg} IgD^{neg} and IgM^{hi} $IgD^{lo/neg}$ B cells in thymus of control mice were gated to show CD43 and CD5 expression. Cells expressing the B-1a phenotype ($CD5^{+}$ $CD43^{+}$) in IgM^{neg} IgD^{neg} and IgM^{hi} $IgD^{lo/neg}$ B cells were defined.

(E) The upper FACS plot shows $CD95^{+}$ $CD38^{lo}$ and $CD95^{neg}$ $CD38^{+}$ subsets in IgM^{neg} B-1a cells from CKO mice. The lower histograms show the data that compare the expression levels of the surface MHC II (IA^{d}), intracellular H2-DM and its inhibitor H2-DO between $CD95^{+}$ $CD38^{lo}$ IgM^{neg} B-1a and $CD95^{neg}$ $CD38^{+}$ IgM^{neg} B-1a cells.

(F) The upper FACS plots show GC B ($CD19^{+}$ $CD95^{+}$ $CD38^{neg}$), $CD95^{+}$ $CD38^{lo}$ IgM^{neg} B-1a and $CD95^{neg}$ $CD38^{+}$ IgM^{neg} B-1a cells in CKO mice. The lower FACS histograms show the data that compare the surface CD38, GL7, PNA and CD150 expression levels among GC B (green line), $CD95^{+}$ $CD38^{lo}$ IgM^{neg} B-1a (red line) and $CD95^{neg}$ $CD38^{+}$ IgM^{neg} B-1a cells (blue line).

(G) The left FACS plot shows that GC B cells ($CD19^{+}$ $CD95^{+}$ $CD38^{neg}$) in CKO mice are further divided into dark zone (DZ) GC B ($CXCR4^{+}$ $CD86^{lo}$) and light zone (LZ) GC B cells ($CXCR4^{lo}$ $CD86^{hi}$). The right FACS histograms show the intracellular H2-DM and H2-DO expression levels expressed by $CD95^{+}$ $CD38^{lo}$ IgM^{neg} B-1a (red line), LZ GC B (blue line) and DZ GC B cells (black line) from CKO mice.

(H) Three representative examples show that the IgH transcripts from IgM^{neg} B-1a cells contain mutations in their V_H region. Obtained sequence (upper line) is aligned with the reference sequence (lower line). The mutated nucleotides are highlighted in red.

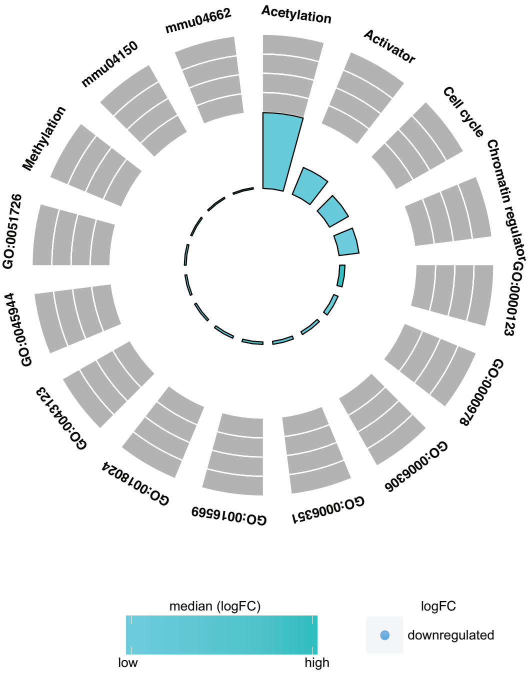

ID	Description
Acetylation	Acetylation
Activator	Activator
Cell cycle	Cell cycle
Chromatin regulator	Chromatin regulator
GO:0000123	histone acetyltransferase complex
GO:0000978	RNA polymerase II core promoter proximal region sequence−specific DNA binding
GO:0006306	DNA methylation
GO:0006351	transcription, DNA−templated
GO:0016569	covalent chromatin modification
GO:0018024	histone−lysine N−methyltransferase activity
GO:0043123	positive regulation of I−kappaB kinase/NF−kappaB signaling
GO:0045944	positive regulation of transcription from RNA polymerase II promoter
GO:0051726	regulation of cell cycle
Methylation	Methylation
mmu04150	mTOR signaling pathway
mmu04662	B cell receptor signaling pathway

Supplementary Figure 6: IgM^{neg} B-a cells in CKO mice down-regulate transcriptional activation programs.

Circular visualization graph shows the selected GO terms for significantly down-regulated genes in IgM^{neg} B-1a cells compared to IgM^{hi} B-1a cells from CKO mice. Each dot in the outer circle represents an individual down-regulated gene. Genes of the same functional term are grouped together. The detail of each group is shown at the right table. In the inner circle, the colored bars summarize the median fold change of the genes sharing the same functional term.

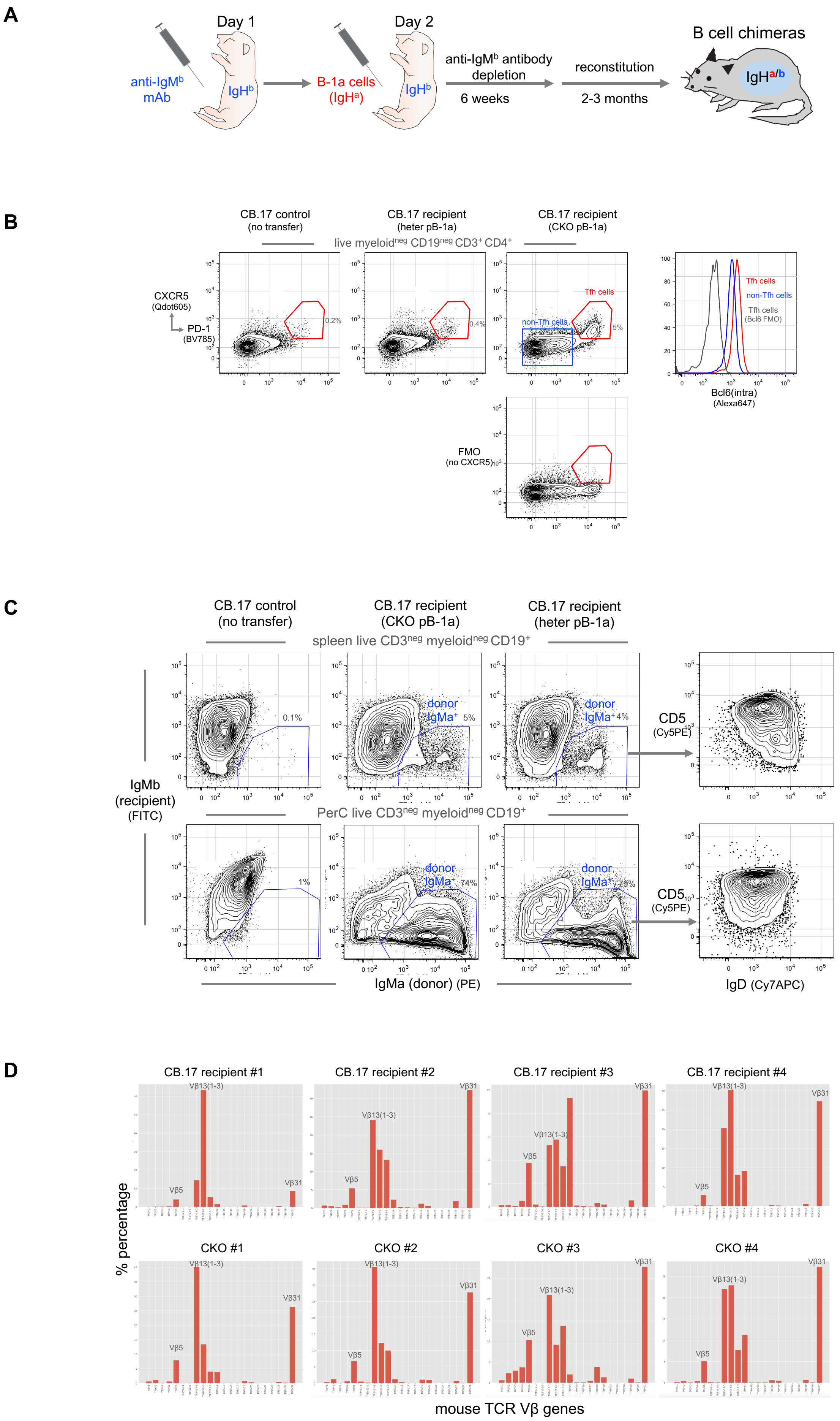
A
Day 1
anti-IgMb mAb
IgHb
Day 2
B-1a cells (IgHa)
IgHb
anti-IgMb antibody depletion
6 weeks
reconstitution
2-3 months
B cell chimeras
IgHa/b
B
CB.17 control (no transfer)
CB.17 recipient (heter pB-1a)
CB.17 recipient (CKO pB-1a)
live myeloidneg CD19neg CD3+ CD4+
CXCR5 (Qdot605)
PD-1 (BV785)
0.2%
0.4%
Tfh cells
non-Tfh cells
5%
FMO (no CXCR5)
Tfh cells
non-Tfh cells
Tfh cells (Bcl6 FMO)
Bcl6(intra) (Alexa647)
C
CB.17 control (no transfer)
CB.17 recipient (CKO pB-1a)
CB.17 recipient (heter pB-1a)
spleen live CD3neg myeloidneg CD19+
0.1%
donor IgMa+ 5%
donor IgMa+ 4%
CD5 (Cy5PE)
IgMb (recipient) (FITC)
PerC live CD3neg myeloidneg CD19+
1%
donor IgMa+ 74%
donor IgMa+ 79%
CD5 (Cy5PE)
IgMa (donor) (PE)
IgD (Cy7APC)
D
CB.17 recipient #1
CB.17 recipient #2
CB.17 recipient #3
CB.17 recipient #4
Vβ13(1-3)
Vβ5
Vβ31
CKO #1
CKO #2
CKO #3
CKO #4
% percentage
mouse TCR Vβ genes

E

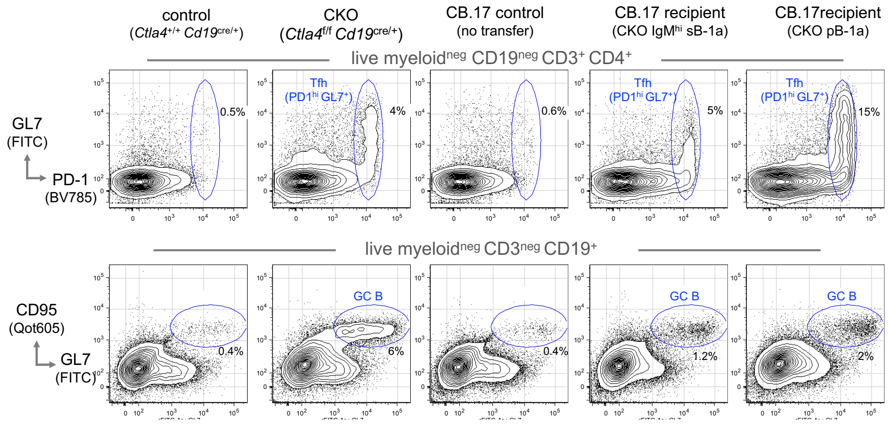

F

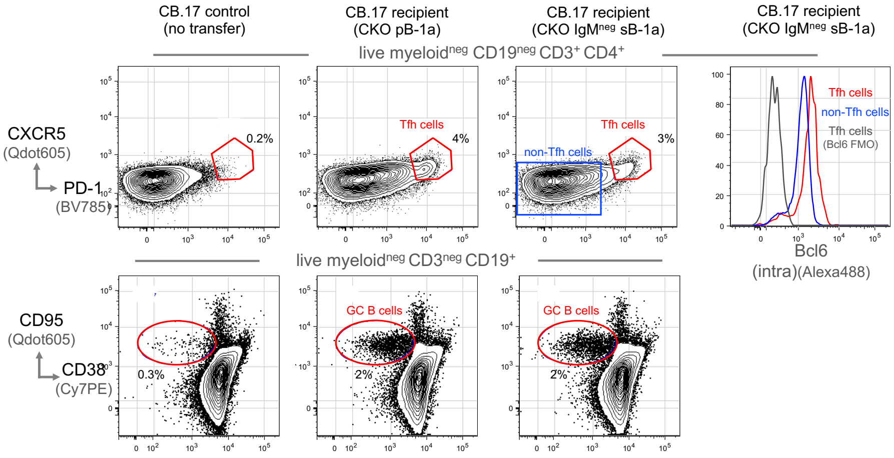

Supplementary Figure 7: When transferred to and reconstituted in IgH allotype congenic recipients, CTLA-4-deficient IgM^{hi} B-1a cells and their differentiated IgM^{neg} descendants induce Tfh cells and GCs in recipient spleen.

(A) The schematic diagram shows the strategy to generate IgH allotype congenic B cell chimera.

(B) Live CD4^{+} T cells in spleen of CB.17 recipients were gated to show Tfh cells (PD-1^{hi} CXCR5^{+}) and non-Tfh cells (PD-1^{neg} CXCR5^{neg}). The rightmost histogram showing the intracellular Bcl6 expression in Tfh (red line) and non-Tfh cells (blue line) in CB.17 recipient reconstituted with CTLA-4-deficient peritoneal B-1a (pB-1a). Two FMO staining, in which the fluorescent anti-CXCR5 or anti-Bcl6 antibody was omitted from the staining cocktail, were used to define CXCR5 and Bcl6-expressing cells, respectively.

(C) Live CD19^{+} B cells from the spleen and PerC of the indicated CB.17 recipients were gated to reveal donor-derived IgMa^{+} B cells, which were further gated to show IgD and CD5 expression.

(D) Graphs show the TCR Vβ gene usage for each Tfh cell sample from CKO mice or CB.17 recipients reconstituted with CTLA-4-deficient B-1a cells. *x*-axis shows mouse TCR Vβ genes; *y*-axis shows the frequency of the TCRβ transcripts expressing each Vβ gene.

(E) Splenic B and T cells from indicated mice were analyzed by FACS. Live CD3^{+} CD4^{+} T cells were gated to show PD-1 and GL7 expression (upper FACS plots) and live CD19^{+} B cells were gated to show CD95 and GL7 expression (lower FACS plots). Tfh cells, PD-1^{hi} GL7^{+}; GC B cells, CD95^{+} GL7^{+}.

(F) Splenic B and T cells from indicated mice were analyzed by FACS. Around 5 x10^{6} of peritoneal B-1a (pB-1a) and 2 x10^{5} of IgM^{neg} splenic B-1a (sB-1a) cells were transferred. Tfh cells, PD-1^{hi} CXCR5^{+}; GC B cells, CD95^{+} CD38^{neg}. The rightmost histogram shows the intracellular Bcl6 expression in Tfh (red line) and non-Tfh cells (blue line) from the CB.17 recipient reconstituted with splenic IgM^{neg} B-1a from CKO mice.

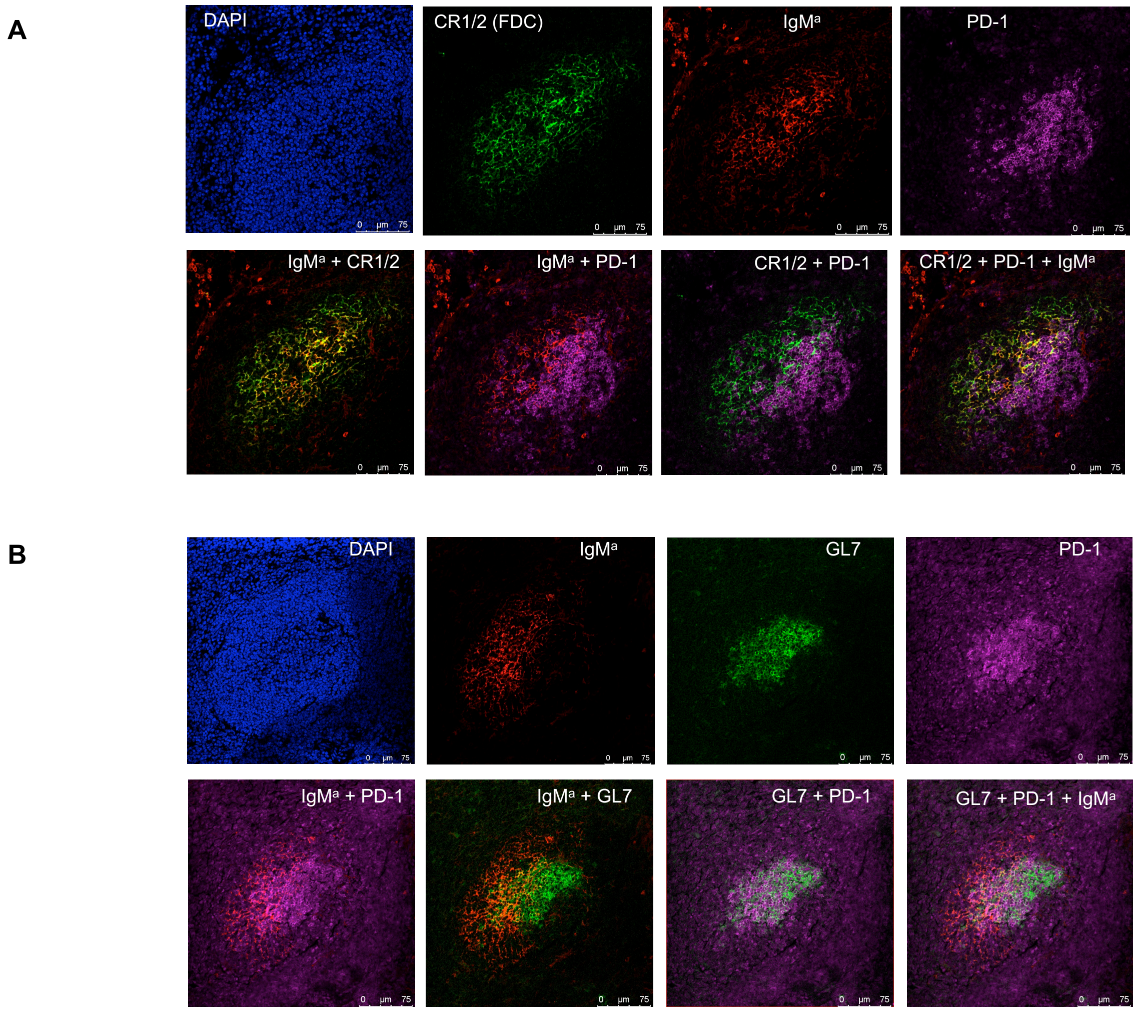

Supplementary Figure 8: CTLA-4 deficient B-1a cells are localized in close association with FDCs and interact with Tfh cells in recipient spleen.

Spleen sections of recipients that have been received PerC B-1a from CKO mice were analyzed by immunofluorescence confocal microscopy. Representative immunofluorescence images of the section stained with indicated individual reagent are shown in the upper and images of two or three-color composites are shown in the lower.

	CDR3 peptide	V(D)J recombination		CDR3 junction		specificity
				addition	deletion	
1	MRYGNYWYFDV	V11-2 D2-8 J1	7/7	0	0	PtC
2	MRYGSSYWYFDV	V11-2 D1-1 J1	6/7	0	0	PtC
3	MRYDGYYWYFDV	V11-2 D2-9 J1	6/7	TG	0	PtC
4	MRYSNYWYFDV	V11-2 D2-6 J1	4/7	0	0	PtC
5	MRYGTYWYFDV	V11-2 D4-1 J1	2/7	0	0	PtC
6	MRYGSYWYFDV	V11-2 D2-11 J1	2/7	CTCG	V11-2(3) J1(1)	PtC
7	AGDYYGYWYFDV	V12-3 D1-2 J1	3/7	G	0	PtC*
8	AGDRYGYWYFDV	V12-3 D2-9 J1	1/7	GACCGT	J1(1)	PtC*
9	ARPYGNYDAMDY	V2-9 D2-8 J4	1/7	CC G	V2-9(2) J4(6)	PtC*
10	ARDYYGSSYWYFDV	V7-1 D1-1 J1	7/7	0	V7-1(3)	PC
11	ARHYYGSSYYFDY	V5-6-1 D1-1 J2	5/7	0	0	unknown
12	ARHYYGSSYWYFDV	V2-6-2 D1-1 J1	1/7	0	0	unknown
		V5-12-2 D1-1 J1	1/7	0	0	
13	VRHYGSSYFDY	V10S4 D1-1 J2	2/7	C	J2(1)	DNA
14	TRWDY	V6-6 J2	2/7	TGG	J2(8)	unknown
15	ARYYYGSSYAMDY	V7-3 D1-1 J4	2/7	0	V7-3(1)J4(8)	unknown
		V1-53 D1-1 J4	2/7	0	J4(4)	
16	ARDYYGSSWYFDV	V7-3 D1-1 J1	3/7	0	V7-3(1) J1(3)	unknown
		V5-9-4 D1-1 J1	1/7	0	J1(3)	
17	ATGTWFAY	V1-53 D4-1 J3	2/7	0	V1-53(2)	unknown
18	ARYDYDYAMDY	V3-5 D2-4 J4	1/7	0	V3-5(2) J4(3)	unknown
19	ASYDGYYWYFDV	V4-1 D2-9 J1	1/7	CTATG	V4-1(3)	
20	ARYYGNYWYFDV	V3-8 D2-8 J1	2/7	0	0	unknown
21	ARRYYGSSYWYFDV	V1-55 D1-1 J1	2/7	AGG		unknown
22	ANWDY	V9-3 D4-1 J2	1/7	0	V9-3(2) J2(8)	unknown
		V14-3 D4-1 J2	5/7	0	V14-3(2) J2(8)	
23	ANWDWYFDV	V14-3 D4-1 J1	2/7	0	V14-3(2) J1(2)	unknown

Supplementary Table 1: The IgH repertoires of IgM^{hi} B-1a and IgM^{neg} B-1a from the same CKO mice co-express the highly conserved V(D)J IgH sequences that are identified to be selected into and shared by in B-1a repertoire of C57Bl/6J mice.

Column 1, the list of CDR3 peptides; Column 2, for each CDR3 peptide, their encoded V(D)J sequence; Column 3, out of 7 CKO mice, number of mice in which this particular V(D)J sequence are found in both IgM^{hi} B-1a and IgM^{neg} B-1a cells; Column 4 and 5, nucleotides added or deleted in CDR3 junctions; Column 6, the specificity for each V(D)J IgH sequence.

CDR3 peptide (PtC-binding) (example 1)	V(D)J	CKO mice	B-1a subset	IgH transcript number	
MRYGNYWYFDV	V11-2 D2-8 J1	A6	IgM^{hi}	IgM	2789
			IgM^{neg}	IgM	1695
				IgD	25
				IgG1	3
				IgE	3
				IgG3	2
				IgG2b	1
		H2	IgM^{hi}	IgM	2206
			IgM^{neg}	IgM	5065
				IgD	36
				IgG2b	5
				IgA	5
				IgG1	4
				IgG3	3
		C11	IgM^{hi}	IgM	2913
				IgD	4
			IgM^{neg}	IgM	1662
				IgD	18
				IgG3	16
				IgG2c	5
				IgG1	3
				IgG2b	2
				IgE	1

CDR3 peptide (T15id PC-binding)	V(D)J	CKO mice	B-1a subset	IgH transcript number	
ARDYYGSSYWYFDV	V7-1 D1-1 J1	H2	IgM^{hi}	IgM	1206
				IgD	18
				IgG3	1
			IgM^{neg}	IgG3	470
				IgA	457
				IgG2c	136
				IgM	133
				IgG2b	70
				IgG1	3
		K5	IgM^{hi}	IgM	671
			IgM^{neg}	IgG3	74
				IgG2b	28
				IgA	23
				IgG2c	11
		C11	IgM^{hi}	IgM	7720
				IgD	8
			IgM^{neg}	IgG3	2706
				IgG2b	719
				IgM	562
				IgG2c	540
				IgA	500
				IgG1	25
				IgD	3

Supplementary Table 2: For the prototypic B-1a V(D)J sequences specific to PtC and PC, IgM is the dominant isotype for IgM^{hi} B-1a cells whereas the class-switched Ig are detected in IgM^{neg} B-1a from the same CKO mice.

Column 1, the list of CDR3 peptides; Column, its encoded V(D)J sequence; Column 3-6, number of Ig isotype transcripts detected in IgM^{hi} B-1a and IgM^{neg} B-1a from indicated CKO mice.

sample	sequence ID	mouse	T cell subset	D50	total nucleotide sequences	distinct CDR3 nucleotide sequences	distinct peptide sequences
1	175454	CB.17 recipient	non-Tfh CD4	**10.3**	1040788	17819	15932
2	175453	C57Bl/6J	non-Tfh CD4	**11.3**	974467	19042	17102
3	175451	CB.17 recipient	non-Tfh CD4	**12.4**	971120	16471	14734
4	175449	CB.17 recipient	non-Tfh CD4	**12.6**	949979	19407	17411
5	175447	CB.17 recipient	non-CD4	**11.4**	862509	18798	17015
6	175446	control	non-Tfh CD4	**10**	852666	14285	12864
7	174325	CKO	Tfh	**1.6**	1741325	7063	5956
8	174319	CKO	Tfh	**1.8**	634281	5057	4365
9	175450	CKO	Tfh	**1.8**	1092535	5330	4542
10	175333	CKO	Tfh	**3.3**	451018	2675	2194
11	174327	CB.17 recipient	Tfh	**1.6**	1955026	6996	5694
12	174324	CB.17 recipient	Tfh	**0.7**	1591853	5003	4093
13	174323	CB.17 recipient	Tfh	**1.4**	991980	4673	3943
14	175445	CB.17 recipient	Tfh	**2.3**	872601	5223	4259

Supplementary Table 3: TCRβ sequencing summary of non-Tfh T cells and Tfh cells.

Phenotypically defined T cell subsets were sorted from indicated mice. The TCRβ cDNA library was generated from each subset and sequenced. Total of 14 separately prepared TCRβ libraries were sequenced with each derived from about $2x10^4$ sorted T subset from an individual mouse. Tfh cells ($CD19^{neg}$ $myeloid^{neg}$ $CD3^+$ $CD4^+$ $PD\text{-}1^{hi}$ $CXCR5^+$), non-Tfh CD4 T cells ($CD19^{neg}$ $myeloid^{neg}$ $CD3^+$ $CD4^+$ $PD\text{-}1^{neg}$ $CXCR5^{neg}$), non CD4 T cells ($CD19^{neg}$ $myeloid^{neg}$ $CD3^+$ $CD4^{neg}$ $PD\text{-}1^{neg}$ $CXCR5^{neg}$). CB.17 recipients refer the chimeric mice reconstituted with CTLA-4-deficient B-1a cells from CKO mice. D50 value refers the D50 metric analysis quantifying the TCRβ CDR3 nucleotide sequence diversity for each T cell sample.

	TCRβ CDR3 peptide	Vβ(Dβ)Jβ recombination	CKO mice (n=4)	CB.17 recipients (n=4)
1	AWSLWGGSQNTLY	V31 (D2) J2-4	2/4	4/4
2	AWSLRGSQNTLY	V31-J2-4	4/4	2/4
3	AWSLGQSGNTLY	V31 (D1) J1-3	3/4	3/4
4	ASSQGQQDTQY	V5 (D1) J2-5	1/4	4/4
5	ASSQKDTQY	V5-J2-5	4/4	2/4
6	ASSQDWGGYAEQF	V5 (D2) J2-1	1/4	4/4
7	ASSQDWGAYAEQF	V5 (D2) J2-1	1/4	4/4
8	ASSQDGENTLY	V5 (D2) J2-4	3/4	2/4
9	ASSPGQQDTQY	V5 (D1) J2-5	2/4	4/4
10	ASSPGQNYAEQF	V5 (D1) J2-1	3/4	2/4
11	ASSPGQGGTEVF	V5 (D1) J1-1	3/4	2/4
12	ASSGGHQNTLY	V13-1 (D1) J2-4	3/4	3/4
13	ASRTGGAETLY	V13-1 (D1) J2-3	2/4	3/4
14	ASGDNSGNTL	V13-2 (D1) J1-3	2/4	4/4
15	ASGLGSQNTLY	V13-2 (D2) J2-4	2/4	3/4
16	ASGGQNTEVF	V13-2 (D1) J1-1	1/4	4/4
17	ASGDVWGQDTQY	V13-2 (D2) J2-5	2/4	3/4
18	ASGDAWGVYEQY	V13-2 (D2) J2-7	3/4	2/4
19	ASSDWGALQNTLY	V13-3 (D2) J2-4	3/4	2/4
20	ASRTGGQNTLY	V13-3 (D2) J2-4	3/4	3/4
21	ASSYQGNTEVF	V4 (D1) J1-1	2/4	4/4
22	ASSLQGNTEVF	V4 (D1) J1-1	1/4	4/4
23	ASSLGNSGNTLY	V15-J1-3	2/4	2/4
24	ASSRQANTEVF	V15 (D1) J1-1	2/4	3/4
25	ASSIGGSNERLF	V19 (D2) J1-4	2/4	1/4

Supplementary Table 4: TCRβ repertoires expressed by Tfh cells from CKO mice and Tfh cells from the recipients reconstituted with CTLA-4 deficient B-1a cells contain the identical Vβ(Dβ)Jβ recombination sequences.

The list of selected TCRβ CDR3 peptide sequences (column 2) encoded by the Vβ(Dβ)Jβ recombinations (column 3) that are shared by TCRβ repertoires expressed by Tfh cells from CKO mice and CB.17 recipients that are reconstituted with CTLA-4-deficient B-1a cells. Column 4 and 5 showing out of four CKO mice and four CB.17 recipients, numbers of mice in which each Vβ(Dβ)Jβ recombination sequence is found.

chimeric mice	donor cells	% IgG1 (GC B cells)	IgG1+ GC B		IgG1neg GC B
			**IgG1a** (MFI)	**IgG1b** (MFI)	**IgG1b** (MFI)
CB.17 recipient 1	pB-1a (CKO)	42%	958	113	109
CB.17 recipient 2	pB-1a(CKO)	44%	911	104	110
CB.17 recipient 3	pB-1a(CKO)	26%	1073	97	102
CB.17 recipient 4	pB-1a(CKO)	35%	978	99	114
CB.17 recipient 5	pB-1a (CKO)	41%	999	101	99
Mean ± SD		37.6 ± 7.3	983 ± 26	102 ± 2.8	106 ± 2.7

Supplementary Table 5: Summary of the FACS analysis of CB.17 chimeric mice reconstituted with peritoneal B-1a (pB-1a) from CKO mice

Column 3 showing the percentage of the induced GC B cells ($CD19^{+}$ $CD95^{+}$ $GL7^{hi}$ $CD38^{neg}$) that express IgG1 on surface in each mouse. Column 4-6 showing MFI values of IgG1-expressing ($IgG1^{+}$) and IgG1-negative GC B cells stained with anti-IgG1a and anti-IgG1b monoclonal antibodies labeled with the same fluorochrome. The mean and standard deviation (SD) are determined for respective measured parameters.

Targeted gene allele	Forward	Reverse
Ctla4 (flox)	GAAACTCTCTCAGGAGGTTGATGC	AGACTGCCTTGGGAAAAGCG
Ctla4 (wild type)	GAGCAAGATGGTGAGTGTGATG	TGTCAAACATGCTCACGTACAG
Cd19 (mutant)	GCGGTCTGGCAGTAAAAACTATC	GTGAAACAGCATTGCTGTCACTT
Cd19 (wild type)	CCTCTCCCTGTCTCCTTCCT	TGGTCTGAGACATTGACAATCA

Supplementary Table 6: Table lists the primers used in mouse genotyping PCR assay

ID#	Glyco-antigens	concentrations (mg/ml)	references
1, 2	Orosomucoid	0.05, 0.25	1,2
3, 4	Asialo-orosomucoid	0.05, 0.25	1,2
5, 6	Agalacto-orosomucoid	0.05, 0.25	1,2
7, 8	Man1Gn2Asn-BSA	0.05, 0.25	3,4
9, 10	Man2Gn2Asn-BSA	0.05, 0.25	3,4
11, 12	Man3Gn2Asn-BSA	0.05, 0.25	3,4
13, 14	Man4Gn2Asn-BSA	0.05, 0.25	3,4
15, 16	Man5Gn2Asn-BSA	0.05, 0.25	3,4
17, 18	Man6Gn2Asn-BSA	0.05, 0.25	3,4
19, 20	Man7Gn2Asn-BSA	0.05, 0.25	3,4
21, 22	Man8Gn2Asn-BSA	0.05, 0.25	3,4
23, 24	Man9Gn2Asn-BSA	0.05, 0.25	3,4
25, 26	Man9Gn2Asn-KLH	0.05, 0.25	5,6
27, 28	Man9Gn2Asn-PAA	0.05, 0.25	This report
29, 30	[(Man9Gn2Asn)4]-TH	0.05, 0.25	6,7
31, 32	Glc1Man9Gn2Asn-BSA	0.05, 0.25	This report
33, 34	Glc1Man9Gn2Asn-RB	0.05, 0.25	This report
35, 36	GlcNAc-RB	0.05, 0.25	This report
37, 38	OSM-Tn	0.05, 0.25	1,8
39, 40	Asialo-PSM (T)	0.05, 0.25	1,8
41, 42	OG 10% 2X (Ii, O-Cores)	0.05, 0.25	1,8
43, 44	TijII (Le^a, I, and O-Cores)	0.05, 0.25	1,8
45, 46	Chondroitin-SO4 A	0.05, 0.25	1
47, 48	Chondroitin-SO4 B	0.05, 0.25	1
49	Chondroitin-SO4 C	0.25	1
Bg	Array background value (N = 48)		

1 Wang, D. *et al.* Carbohydrate microarrays for the recognition of cross-reactive molecular markers of microbes and host cells. *Nat Biotechnol* **20**, 275-281(2002).

2 Wang, D. *et al.* Glycan arrays lead to the discovery of autoimmunogenic activity of SARS-CoV. *Physiol Genomics* **18**, 245-248 (2004).

3 Toonstra, C. *et al*. Top-Down Chemoenzymatic Approach to Synthesizing Diverse High-Mannose N-Glycans and Related Neoglycoproteins for Carbohydrate Microarray Analysis. *Bioconjug Chem* **29**, 1911-1921 (2018).

4 Feng, M. *et al.* Programmed cell removal by calreticulin in tissue homeostasis and cancer. *Nat Commun* **9**, 3194 (2018).

5 Newsom-Davis, T. E. *et al.* Enhanced immune recognition of cryptic glycan markers in human tumors. *Cancer research* **69**, 2018-2025 (2009).

6 Wang, D. *et al.* Uncovering cryptic glycan markers in multiple sclerosis (MS) and experimental autoimmune encephalomyelitis (EAE). *Drug development research* **75**, 172-188 (2014).

7 Wang, D. *et al.* Anti-oligomannose antibodies as potential serum biomarkers of aggressive prostate cancer. *Drug Dev. Res.* **74**, 65-80 (2013).

8 Wang, D. *et al*. Carbohydrate microarrays identify blood group precursor cryptic epitopes as potential immunological targets of breast cancer. *J Immunol Res* **2015**, 510810 (2015).

Supplementary Table 7: Table lists the full name, concentration and the reference of the glycan antigens shown in carbohydrate microarray analysis (Figure 4d).
