# Supplementary material for: CTLA-4 expression by B-1a B cells is essential for immune tolerance
Source: Nat Commun. 2021 Jan 22;12:525. doi: 10.1038/s41467-020-20874-x (PMC7822855; doi:10.1038/s41467-020-20874-x)
Supplement: Supplementary file 3 — Reporting Summary [file 41467_2020_20874_MOESM3_ESM.pdf]

nature research

Corresponding author(s): Yang Yang
Last updated by author(s): Nov 13, 2020

Reporting Summary

Nature Research wishes to improve the reproducibility of the work that we publish. This form provides structure for consistency and transparency in reporting. For further information on Nature Research policies, see Authors & Referees and the Editorial Policy Checklist .

Statistics

For all statistical analyses, confirm that the following items are present in the figure legend, table legend, main text, or Methods section.

n/a	Confirmed	
☐	☒	The exact sample size (*n*) for each experimental group/condition, given as a discrete number and unit of measurement
☐	☒	A statement on whether measurements were taken from distinct samples or whether the same sample was measured repeatedly
☐	☒	The statistical test(s) used AND whether they are one- or two-sided<br>*Only common tests should be described solely by name; describe more complex techniques in the Methods section.*
☒	☐	A description of all covariates tested
☒	☐	A description of any assumptions or corrections, such as tests of normality and adjustment for multiple comparisons
☐	☒	A full description of the statistical parameters including central tendency (e.g. means) or other basic estimates (e.g. regression coefficient) AND variation (e.g. standard deviation) or associated estimates of uncertainty (e.g. confidence intervals)
☐	☒	For null hypothesis testing, the test statistic (e.g. *F*, *t*, *r*) with confidence intervals, effect sizes, degrees of freedom and *P* value noted<br>*Give P values as exact values whenever suitable.*
☒	☐	For Bayesian analysis, information on the choice of priors and Markov chain Monte Carlo settings
☒	☐	For hierarchical and complex designs, identification of the appropriate level for tests and full reporting of outcomes
☒	☐	Estimates of effect sizes (e.g. Cohen's *d*, Pearson's *r*), indicating how they were calculated

Our web collection on statistics for biologists contains articles on many of the points above.

Software and code

Policy information about availability of computer code

Data collection	For flow cytometry analysis, cells were analyzed or sorted on FACSAria IIu (BD Biosciences) at Stanford Shared FACS Facility and the data were required using FACS Diva Software (version 8.0, BD Bioscience); mouse IgH and TCR beta chain repertoire data was collected by Illumina paired-end sequencing on Illumina MiSeq platform in HudsonAlpha Institute for Biotechnology; RNA-seq data was collected by Illumina HiSeq 4000 at the Genome Sequencing Service Center by Stanford Center for Genomics and Personalized Medicine Sequencing; For carbohydrate microarray analysis, mouse sera samples were scanned using t ScanArray5000A Microarray Scanner (version 4.0, PerkinElmer Life Science); Immunofluorescence confocal microscopy imaging data was collected on Leica SP5 confocal machine in Stanford Cell Sciences Imaging Facility for Immunofluorescence confocal microscopy analysis.
Data analysis	FACS data was analyzed by Flowjo software version 9.7 (www.flowjo.com); For mouse IgH and TCR beta chain repertoire analysis, sequence reads were de-multiplexed according to barcode sequences at the 5' end of reads from the constant region. Reads were then trimmed according to their base qualities with a 2-base sliding window, if either quality value in this window is lower than 20, this sequence stretches from the window to 3' end were trimmed out from the original read. Trimmed pair-end reads were joined together through overlapping alignment with a modified Needleman-Wunsch algorithm. If paired forward and reverse reads in the overlapping region were not perfectly matched, both forward and reverse reads were thrown out without further consideration. The merged reads were mapped using a Smith-Waterman algorithm to germline V, D, J and C reference sequences downloaded from the IMGT web site (Lefranc, 2003). To define the CDR3 region, the position of CDR3 boundaries of reference sequences from the IMGT database were migrated onto reads through mapping results and the resulting CDR3 regions were extracted and translated into amino acids; Data of carbohydrate microarray analysis were calculated using ScanArray Express software (PerkinElmer Life Science); For RNA-seq analysis, RNA-seq reads were alighned to the mouse reference genome (mm9) using TopHat (version 2.0.13) with the transcript annotation supplied. Both the mouse reference genome and transcript annotation were downloaded from illumina iGenomes. The mapped reads were then assigned to genes using Python package HTseq (Python 2.7, HTseq version 0.6.0), with the default union-counting mode. To perform differential gene expression analysis, DESeq2 R package (R 3.3.1, DEseq 2 version 1.12.4) was applied with an adjusted P-value of 0.05 as the cutoff. Then the gene set annotation enrichment analysis was peformed using The Database for Annotation, Visualization and Integrated Discovery (DAVID). functional annotation tool web sever. Terms with a false discovery rate (FDR) of 10% were considered significant. The GOPlot R package (version 1.0.2) was used to visualize the gene set annotation enrichment analysis results;

Immunofluorescence confocal microscopy imaging data was analyzed using LAS AF 2.7.9723.3 software of Leica Microsystem; We use JMP Pro 14.1.0 software (www. Jmp.com) for most of the statistical analysis.

For manuscripts utilizing custom algorithms or software that are central to the research but not yet described in published literature, software must be made available to editors/reviewers. We strongly encourage code deposition in a community repository (e.g. GitHub). See the Nature Research guidelines for submitting code & software for further information.

Data

Policy information about availability of data

All manuscripts must include a data availability statement. This statement should provide the following information, where applicable:

- Accession codes, unique identifiers, or web links for publicly available datasets
- A list of figures that have associated raw data
- A description of any restrictions on data availability

Data in this study are available within the Article and Supplementary files, or available from the corresponding authors on reasonable request. IgH and TCRβ sequence datasets generated in this study are available at NCBI Sequence Read Archive (SRA) under the accession code RPJNA678122. The RNA-seq dataset is available at Gene Expression Omnibus (GEO) under the accession code GSE161409.

Field-specific reporting

Please select the one below that is the best fit for your research. If you are not sure, read the appropriate sections before making your selection.

[x] Life sciences   [ ] Behavioural & social sciences   [ ] Ecological, evolutionary & environmental sciences

For a reference copy of the document with all sections, see nature.com/documents/nr-reporting-summary-flat.pdf

Life sciences study design

All studies must disclose on these points even when the disclosure is negative.

Sample size	sample sizes are not predetermined through statistical methods. Instead, they are chosen based on the previously reported results such that appropriate statistical testing could yield significant results.
Data exclusions	no data was excluded
Replication	all experiments were independently repeated to ensure the findings are reproducible. The numbers of the experiment times, and the mice used the experiment are indicated in the figure legends.
Randomization	mice were not randomized in the study. Specifically, we assigned mice expressing the different genotype from the same litter into the experiment and control groups. In addition, age-matched mice are used in experimental and control groups. In some experiments, we treated the gender of the animals as an experimental variable and distribute litters appropriately.
Blinding	investigators are not blinded to the group allocation. Because we use inbred mouse strains, no specific randomization or exclusion criteria are applied to mouse samples.

Reporting for specific materials, systems and methods

We require information from authors about some types of materials, experimental systems and methods used in many studies. Here, indicate whether each material, system or method listed is relevant to your study. If you are not sure if a list item applies to your research, read the appropriate section before selecting a response.

Materials & experimental systems

n/a	Involved in the study	
[ ]	[x]	Antibodies
[x]	[ ]	Eukaryotic cell lines
[x]	[ ]	Palaeontology
[ ]	[x]	Animals and other organisms
[x]	[ ]	Human research participants
[x]	[ ]	Clinical data

Methods

n/a	Involved in the study	
[x]	[ ]	ChIP-seq
[ ]	[x]	Flow cytometry
[x]	[ ]	MRI-based neuroimaging

Antibodies

Antibodies used	Fluorochrome-conjugated antibodies include: anti-CD21-FITC (clone 7E9, Biolegend, cat#123408), anti-IAd-FITC(clone AMS-32.1, BD, cat#553547), anti-GL7-FITC (clone GL7, BD, cat553666), anti-IgMb-FITC (clone AF6-78, BD,cat#553520), anti-IgMa-BV785 (clone DS-1, Biolegend, cat#743890), anti-CD43-PE (clone S11, Biolegend, cat#143206), anti-CD138-PE (clone 281-2, Biolegend, cat#142504), anti-CD267-APC (clone eBioBF10-3, eBioscience, cat#17-5942-82), anti-CD38-Alexa488 (clone 90, Biolegend,

cat#102714), anti- H2-M-Alexa488 (clone 2E5A, BD, cat# 552405), anti-CD5-PE-Cy5 (clone 53-7.3, Biolegend, cat#100610), anti-CD19-PE-Cy5.5 (clone ID3, Invitrogen, cat#35-0193-82), anti-IgG1-PE-Cy7(clone RMG1-1, Biolegend, cat#406614), anti-ICOS-PE-Cy7(clone C398.4A, Biolegend, cat#313520), anti-CD150-PE-Cy7(clone TC150-12F12.2, Biolegend, cat#115913), anti-IgM-PE-Cy7 (clone R6-60.2, BD,cat#552867), anti-B220-Alexa700(clone RA3-6B2, Biolegend, cat#103232), anti-CD3-Alexa700(clone 145-2C11, Biolegend, cat#100216), anti-IgM-APC (clone RMM1, Biolegend, cat#406509), anti-Bcl6-APC (clone 7D1, Biolegend, cat#648305), anti-Ki67-Alexa647 (clone 11F6, Biolegend, cat#151206), anti-CTLA-4-PE(clone UC10-4F10-11, BD, cat#553720), PE Hamster IgG1 isotype control (clone A19.3, BD, cat#553972), anti-CTLA-4-APC (clone UC10-4F10-11, BD, cat#564331) anti-Foxp3-APC (clone FJK-16S, eBioscience, cat#17-5773-80), anti-Foxp3-PE (clone FJK-16S, eBioscience, cat#12-5773-82), anti-IgD-APC-Cy7 (clone 11-26c.2a, Biolegend, cat#405716), anti-CD4-APC-Cy7(clone GK1.5, BD,cat#552051), anti-CD23-biotin (clone, B3B4, Biolegend, cat#101604), anti-CD80-biotin (clone 16-10A, BD, cat#553767), anti-CD86-biotin (clone GL-1, Biolegend, cat#105004), anti-CD95-Qdot605 (clone SA367H8, Biolegend, cat#152612), anti-IgG1a-biotin (clone 10.9, BD, cat#553500), anti-IgG1b-biotin (clone B68.2, BD, cat#553533), anti-CXCR5-Brilliant BV605 (clone L138D7, Biolegend, cat#145513), anti-CD11b-PB (clone M1/70, Biolegend, cat#101224), anti-Gr-1-PB(clone RB6-8C5, Biolegend,ca#108430), anti-TCRαβ-PB(clone H57, Invitrogen,cat#HM3628), anti-CD11c-PB (clone N418, Biolegend, cat#117322), anti-CD3ε-PB (clone 145-2C11, Biolegend, cat#100334), anti-F4/80-PB (clone BM8, Biolegend, cat#123124), anti-CD279 (PD-1)-Brilliant violet 785 (clone 29F.1A12, Biolegend, cat#135225), anti-CD25-PE(clone 7D4, BD, cat#558642). LEAF purified anti-CD154 (clone MR1, Biolegend, cat#106508), anti-CD4 (clone GK1.5, Biolegend, cat#100442), IgG isotype control (clone SHG-1, Biolegend, cat#70647), anti-IgMb (clone AF6-78, customer produced by Biolegend).

Validation

Validation statements for flow are included in the Manufacturers' website

Animals and other organisms

Policy information about studies involving animals; ARRIVE guidelines recommended for reporting animal research

Laboratory animals

BALB/c (IgHa), CB.17 (IgHb), C57BL/6J and TCR beta,delta chain double knockout (C57BL/6J background) mice are purchased from Jackson laboratory. Eμ-TCL1 (C57BL/6J) transgenic mice are generously provided by Dr. Thomas Kipps at the UC San Diego Moores Cancer Center. BALB/c mice expressing a floxed CTLA4 gene (CTLA4fl/fl) were provided by Dr. Shimon Sakaguichi (Kyoto University). Homozygous CD19cre mice (CD19tm1(cre)cgn) were purchased from the Jackson Laboratory. Both female and male of each mouse strain are used. Information of the mouse age has been indicated in legends of Figure and Supplementary figures. Mice were housed in specific pathogen-free facilities and given standard chow and acidified water.

Wild animals

no wild animals were used in this study.

Field-collected samples

none

Ethics oversight

mice were breed, maintained and experimentally conducted under the protocols approved by Stanford Veterinary Service Center.

Note that full information on the approval of the study protocol must also be provided in the manuscript.

Flow Cytometry

Plots

Confirm that:

- [x] The axis labels state the marker and fluorochrome used (e.g. CD4-FITC).
- [x] The axis scales are clearly visible. Include numbers along axes only for bottom left plot of group (a 'group' is an analysis of identical markers).
- [x] All plots are contour plots with outliers or pseudocolor plots.
- [x] A numerical value for number of cells or percentage (with statistics) is provided.

Methodology

Sample preparation

Single cell suspension from the spleen, lymph node, peritoneal cavity and thymus was prepared. To obtain the thymocytes, the connective tissues attached to thymic lobes were carefully peeled off to remove the parathymic lymph nodes. About 1.25 x 10^6 cells were incubated with LIVE/DEAD Aqua (cat# L34957, Invitrogen, ThermoFisher, Inc.), washed, and incubated with anti-CD16/CD32 (FcRII/III) mAb to block Fc-receptors. Cells were then stained on ice for 20 minutes with a “cocktail” of fluorochrome-conjugated antibodies. For second step staining, after washing, cells were stained with Streptavidin-Qdot 605 (cat# Q10101MP, Invitrogen, ThermoFisher, Inc.). For intracellular staining, after the surface staining, cells were fixed and permeablized with Foxp3/transcription factor staining buffer set (cat#00-5523-00, Invitrogen, ThermoFisher, Inc.), and then stained with fluorochrome-conjugated antibodies. BrdU staining was performed using BrdU flow kit (BD Bioscience). Before staining thymic B cells, single cell suspension cells from thymus are first enriched with B cells by negative selection using mouse pan-B cell isolation kit (StemCell Technology). For FACS sorting of B-1a cells, cells were first enriched for B cells by negative selection using mouse pan-B cell isolation kit (Cat#19844, StemCell Technology). For FACS soring of plasma cells, cells were first enriched for CD138+ plasma cells by positive selection using mouse CD138 positive selection kit (cat#18957, StemCell Techology). After the enrichment, cells were then stained with surface markers.

Instrument

FACSAria (BD, BioSciences)

Software	data were acquired using Diva (BD, Biosciences) and analyzed using Flowjo software (www.flowjo.com)
Cell population abundance	sorted IgMhi B-1a and IgMneg B-1a populations represent about 1% of CD19+ B cells in spleens of 2-3 monthsold CTLA4 B cell CKO mice; Tfh cells represent about 3% of CD4 T cells of 2-3 months old CKO mice. after sorting, cells were re-analyzed to confirm the sorting purity was over 95%.
Gating strategy	Gating strategy were shown in the figure or supplementary figure and details were indicated in the legends of respected figures. For all FACS analysis and sorting experiments in this study, FSC/SSC is used the starting gate to gate on singlet cells. Live cells are further gated from singlet cells based on LIVE/DEAD Aqua dye. Live B cells or T cells are further gated based on lineage markers. The boundaries between positive and negative stained cell population are defined based on FACS plot generated from cells stained the isotype antibody or fluorescent minus one (FMO) staining, in which the targeting fluorescent antibody is omitted from the staining cocktail.

[x] Tick this box to confirm that a figure exemplifying the gating strategy is provided in the Supplementary Information.
